# Synergistic effects of vorinostat (SAHA) and azoles against *Aspergillus* species and their biofilms

**DOI:** 10.1186/s12866-020-1718-x

**Published:** 2020-02-07

**Authors:** Bo Tu, Gendi Yin, Hui Li

**Affiliations:** 1 Department of Otorhinolaryngology and Head Neck Surgery, the First Affiliated Hospital of Jinan University, Guangzhou, 510630 Guangdong People’s Republic of China; 2 Department of Otolaryngology Head Neck Surgery, The Third Affiliated Hospital of Sun Yat-sen University, Guangzhou, 510630 Guangdong People’s Republic of China

**Keywords:** *Aspergillus*, Biofilm, SAHA, Combination, Antifungals, *HSP90*

## Abstract

**Background:**

Invasive aspergillosis is a fungal infection that occurs mainly in immunocompromised patients. It is responsible for a high degree of mortality and is invariably unresponsive to conventional antifungal treatments. Histone deacetylase inhibitors can affect the cell cycle, apoptosis and differentiation. The histone deacetylase inhibitor vorinostat (SAHA) has recently received approval for the treatment of cutaneous T cell lymphoma. Here, we investigated the interactions of SAHA and itraconazole, voriconazole, and posaconazole against *Aspergillus* spp*.* in vitro using both planktonic cells and biofilms.

**Results:**

We investigated 20 clinical strains using broth microdilution checkerboard methods. The results showed synergy between SAHA and itraconazole, voriconazole, and posaconazole against 60, 40, and 25% of tested isolates of planktonic *Aspergillus* spp., respectively. Similar synergy was also observed against *Aspergillus* biofilms. The expression of the azole-associated multidrug efflux pumps *MDR1*, *MDR2*, *MDR3* and *MDR4,* as well as that of *HSP90,* was measured by RT-PCR. The results indicated that the molecular mechanism of the observed synergistic effects in *Aspergillus fumigatus* may be partly associated with dampened expression of the efflux pump genes and, furthermore, that *HSP90* suppression may be a major contributor to the observed synergistic effects of the drugs.

**Conclusions:**

SAHA has potential as a secondary treatment to enhance the effects of azoles against both biofilm and planktonic cells of *Aspergillus* spp*.* in vitro*.* This effect occurs mostly by inhibition of *HSP90* expression.

## Background

In recent years, an increased incidence of invasive aspergillosis (IA) has affected the lives of many immunocompromised patients [1]. It has been reported that some *Aspergillus* strains are resistant to azoles, polyenes and echinocandins, thus reducing the effectiveness of treatments [2]. Combinations of azoles and echinocandins against *Aspergillus* or azole-resistant *Aspergillus* are known to be effective for the treatment of serious infections [2, 3]. Moreover, new imidazoles, such as lanoconazole and luliconazole, strongly inhibit the growth of *Aspergillus spp.* [4, 5]. An additional challenge is the presence of *Aspergillus* biofilms, which are thought to contribute to virulence in IA and aspergilloma [6]. In vivo findings show that in sinus aspergillomas, *A. fumigatus* may grow as a typical biofilm characterized by hyphae anchored within an extracellular matrix. Similar biofilms have also been observed on contact lenses in fungal keratitis, in bronchoalveolar lavage fluids of chronic pulmonary aspergillosis and in neutropenic cancer patients with IA [6, 7]. Without adequate therapy, these diseases result in long-term suffering of patients. The minimal inhibitory concentrations (MICs) of antifungals against the biofilm form of *Aspergillus* spp. are predominantly high, particularly among the azoles [6, 8]. Therefore, there is an urgent necessity for new treatment approaches.

Histone deacetylases (HDACs) are enzymes that specifically remove acetyl groups from lysine residues on histones. HDACs also act on other cellular proteins. This deacetylation can affect gene regulation as well as other cellular functions [9]. HDAC inhibitors are able to block the cell cycle, induce apoptosis, and terminate cellular differentiation [9]. It has been reported that combinations of HDAC inhibitors with an azole can reverse fungal resistance to azoles by blocking the HSP90-dependent response [10]. Givinostat, MGCD290 and trichostatin A (TSA) have been reported to act synergistically with azoles against *Candida* and *Aspergillus* in vitro [10–13]. Hence, the combination of HDAC inhibitors and antifungals is a promising approach to address fungal drug resistance. Vorinostat (suberoylanilide hydroxamic acid or SAHA) is a novel HDAC inhibitor that blocks the activity of HDAC1 and HDAC3, as well as causes hyperacetylation of histone H4 [9]. SAHA is an analog of TSA that has an extended half-life and improved oral bioavailability [14]. SAHA has been approved by the FDA as a treatment option for cutaneous T cell lymphoma [15]. Additionally, it has also been shown that SAHA has synergistic effects with proteasome inhibitors such as carfilzomib, inhibiting T cell leukemia/lymphoma cell growth in an in vivo xenograft model [16]. Vorinostat is an antineoplastic drug that has been found to have anticryptosporidial effects in mice, where it acts in a dose-dependent manner against parasite oocysts. The estimated in vivo 50% inhibition dose (ID50) value was approximately 7.5 mg/kg [17]. However, the antifungal potential of SAHA as well as its effects in combination with azoles are still not well understood. The aim of this study was to investigate the effects of SAHA alone as well as in combination with azoles against *Aspergillus* spp. and their biofilms.

## Results

### In vitro interactions of SAHA and azoles against planktonic cells

The ranges of the MICs (100% inhibition, assessed visually) of SAHA, itraconazole (ITR), voriconazole (VRC), and posaconazole (POC) for isolates of planktonic *Aspergillus* spp. were ≥ 16 μg/ml, 1 to 2 μg/ml, 0.5 to 1 μg/ml, and 0.5 to 1 μg/ml, respectively (Table 1). Moreover, when SAHA was combined with ITR, synergistic effects were observed in 66% (8/12) of *A. fumigatus* strains, 60% (3/5) of *Aspergillus flavus* strains and 33% (1/3) of *Aspergillus terreus* strains. Synergistic effects of SAHA and VRC against *A. fumigatus, A. flavus* and *A. terreus* were apparent in 50% (6/12 strains), 20% (1/5 strains) and 33% (1/3 strains) of strains, respectively. When SAHA was combined with POC against planktonic cells, synergistic effects against *A. fumigatus, A. flavus,* and *A. terreus* were observed for 25% (3/12 strains), 20% (1/5 strains), and 33% (1/3 strains) of strains, respectively (Table 1).

**Table 1:** MICs (μg/ml) of the drugs alone and FICIs for combinations of SAHA with azoles against planktonic *Aspergillus* spp.

Strain	SAHA	ITR	SAHA/ITR	FICI	VRC	SAHA/VRC	FICI	POC	SAHA/POC	FICI
*A. fumigatus*										
AF293	≥16	2	4/0.5	0.38	1	2/0.25	0.31	1	2/0.25	0.31
AF102	≥16	1	2/0.31	0.56	1	2/0.5	0.56	1	2/0.5	0.56
AF103	≥16	2	2/0.5	0.31	1	2/0.25	0.31	1	4/0.5	0.63
AF104	≥16	2	2/0.5	0.31	1	2/0.25	0.31	1	2/0. 5	0.56
AF105	≥16	1	2/1	1.06	1	2/0.5	0.56	1	2/0.25	0.31
AF106	≥16	2	2/0.5	0.31	1	2/0.25	0.31	1	2/0.5	0.56
AF107	≥16	1	2/0.25	0.31	0.5	2/0.25	0.56	1	2/0.5	0.56
AF108	≥16	1	2/0.25	0.31	1	2/0.125	0.19	1	4/0.5	0.63
AF109	≥16	2	2/0.5	0.31	1	2/0.25	0.31	1	2/1	1.06
AF1010	≥16	2	2/2	0.56	0.5	2/0.25	0.56	0.5	2/0.5	1.06
AF1011	≥16	1	2/0.5	0.56	1	2/0.5	0.56	1	2/0.25	0.31
AF1012	≥16	1	2/0.5	0.56	1	2/1	1.06	1	2/0.5	0.56
*A. flavus*										
AFLA101	≥16	2	2/0.5	0.31	1	2/0.25	0.31	0.5	2/0.5	1.06
AFLA102	≥16	2	2/1	0.56	1	2/0.5	0.56	1	2/0.5	0.56
AFLA103	≥16	2	2/0.5	0.31	1	2/0.25	0.31	1	2/0.25	0.31
AFLA104	≥16	2	2/0.5	0.31	1	1/0.5	0.53	1	2/0.5	0.56
AFLA105	≥16	2	2/1	0.56	1	2/0.25	0.31	1	4/1	1.06
*A. terreus*										
AT101	≥16	2	2/0.5	0.31	0.5	1/0.25	0.53	1	2/0.5	0.56
AT102	≥16	2	2/1	0.56	1	2/0.25	0.31	0.5	2/0.5	1.06
AT103	≥16	1	2/0.5	0.56	1	2/0.25	0.31	1	2/0.25	0.31

FICI results: synergy (FICI of ≤0.5); indifference (no interaction [FICI of > 0.5 to ≤4]). *ITR* itraconazole, *VRC* voriconazole, *POC* posaconazole, *SAHA* suberoylanilide hydroxamic acid

### In vitro interactions of SAHA and azoles against biofilms

The sessile minimal inhibitory concentrations (SMIC_80_) were defined as concentrations resulting in an 80% reduction in the metabolic activity of the biofilm. The SMIC_80_ ranges of SAHA, ITR, VRC, and POC against *Aspergillus* biofilms were ≥ 256 g/ml, ≥256 g/ml, 128 to 256 g/ml, and ≥ 256 g/ml, respectively (Table 2). SMIC values were greater than the MICs for each drug. When SAHA was combined with ITR against *Aspergillus* biofilms, the fractional inhibitory concentration index (FICI) was calculated from the SMIC_80_. Similar synergistic effects were observed against 16 isolates of *Aspergillus* spp., consisting of 11 strains of *A. fumigatus* (11/12), four strains of *A. flavus* (4/5) and one strain of *A. terreus* (1/3). For the SAHA and VRC combination, the FICIs displayed synergistic effects against eight strains of *A. fumigatus* (8/12), five strains of *A. flavus* (5/5) and one strain of *A. terreus* (1/3). For the combination of SAHA and POC, the FICIs revealed synergistic effects against 13 strains. No antagonistic effects were observed in these combinations.

**Table 2:** SMIC80 and FICIs of combinations of SAHA with azoles against *Aspergillus* biofilms

Strain	SMIC_80_ (μg/ml)				SMIC_80_			SMIC_80_		
	SAHA	VRC	SAHA/VRC	FICI	ITR	SAHA/ITR	FICI	POC	SAHA/POC	FICI
AF293	≥256	≥256	128/128	0.50	≥256	128/64	0.38	≥256	128/128	0.50
AF102	≥256	≥256	128/128	0.50	≥256	128/128	0.50	≥256	128/256	0.75
AF103	≥256	128	128/32	0.50	≥256	128/256	0.75	≥256	128/256	0.75
AF104	≥256	≥256	64/64	0.25	≥256	64/64	0.25	≥256	128/128	0.50
AF105	≥256	≥256	128/128	0.50	≥256	64/128	0.38	≥256	64/64	0.25
AF106	≥256	≥256	128/128	0.50	≥256	128/128	0.50	≥256	128/128	0.50
AF107	≥256	128	128/64	0.75	≥256	128/128	0.50	≥256	128/128	0.50
AF108	≥256	256	128/128	0.75	≥256	64/64	0.25	256	128/128	0.75
AF109	≥256	256	64/32	0.25	≥256	128/128	0.50	256	128/128	0.75
AF1010	≥256	128	128/64	0.75	≥256	128/128	0.50	≥256	128/128	0.50
AF1011	≥256	128	64/64	0.63	≥256	128/128	0.50	≥256	128/128	0.50
AF1012	≥256	128	64/32	0.38	≥256	128/128	0.50	≥256	128/256	0.75
AFLA1	≥256	≥256	128/128	0.50	≥256	128/128	0.50	256	128/128	0.75
AFLA2	≥256	≥256	128/128	0.50	≥256	128/128	0.50	≥256	128/128	0.50
AFLA3	≥256	≥256	128/128	0.50	≥256	128/128	0.50	≥256	64/64	0.25
AFLA4	≥256	≥256	128/64	0.38	≥256	128/128	0.50	≥256	128/128	0.50
AFLA5	≥256	≥256	128/128	0.50	≥256	128/256	0.75	≥256	128/128	0.50
AT101	≥256	128	128/128	1.25	≥256	128/256	0.75	≥256	128/128	0.50
AT102	≥256	128	64/64	0.63	≥256	128/256	0.75	≥256	128/256	0.75
AT103	≥256	≥256	64/64	0.25	≥256	128/128	0.50	≥256	128/128	0.50

FICI results: synergy (FICI of ≤0.5); indifference (no interaction [FICI of > 0.5 to ≤4]). *ITR* itraconazole, *VRC* voriconazole, *POC* posaconazole, *SAHA* suberoylanilide hydroxamic acid

### Triple combinations with TSA

Synergistic interactions were also observed with the triple combinations. After adding 2 μg/ml TSA to each group, the FICIs of the different combinations were no different from those of SAHA combined with azoles only (data were the same as those shown in Table 1). There was also no observed antagonism.

### Quantification of gene expression by RT–PCR

To determine whether the synergistic mechanism of SAHA with azoles against *A. fumigatus* is due to down-regulation of efflux pump genes or *HSP90*, qRT-PCR was performed to analyze the expression levels of these genes under conditions of SAHA or azole treatment alone as well as in combination.

**Fig. 1:**
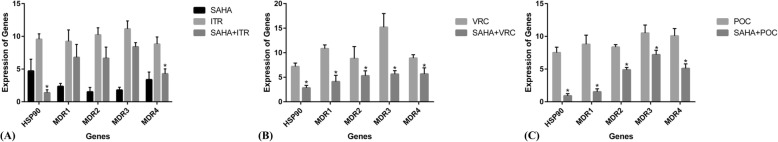
Relative expression levels of the drug efflux pump genes and *HSP90* in AF293 cells after drug treatment. **a** Expression levels of Genes in SAHA, ITR and ITR + SAHA treated groups; **b** Expression levels of Genes in VRC and VRC + SAHA treated groups; **c** Expression levels of Genes in POC and POC + SAHA treated groups. Assays were carried out in triplicate by biological replication and analyzed for statistical significance by multiple t tests using GraphPad Prism Software. The results are significantly different between azoles alone and azoles+SAHA combinations (*: *P* values <0.05). ITR: itraconazole; VRC: voriconazole; POC: posaconazole; SAHA: suberoylanilide hydroxamic acid

As shown in Fig. 1, all the MDR-related efflux pump genes and *HSP90* tested in this study were up-regulated in both the azole-treated and SAHA-only groups. Further assessment indicated that the expression of these genes is suppressed when azoles and SAHA are used in combination. This observation was especially evident in the case of *HSP90*. The expression of *HSP90* was reduced by 6.8-fold in response to treatment with ITR with SAHA, 2.5-fold in response to treatment with ITR with SAHA, and 8.25-fold in response to treatment with ITR with SAHA compared to that with azole-only treatment. Moreover, the attenuated expression of the efflux pump genes was relatively mild in the combination groups compared to that in the azole-alone groups (1.3- to 2.6-fold). Only MDR1 was repressed (by 5.7-fold) when SAHA was combined with POC compared to that with POC alone (*p* < 0.05).

These results show that the synergy of SAHA with azoles may be associated with suppression of *HSP90* expression*.* This finding is consistent with a previously published report on the effects of TSA [9]. However, this synergy has very little effect, if any, on the inhibition of efflux pump genes in *A. fumigatus*.

## Discussion

In *Aspergillus*, HDACs influence virulence by controlling transcription and regulating essential protein functions [9]. The findings of the current study show that the new HDAC inhibitor SAHA is not effective against all planktonic and biofilm isolates of *Aspergillus* spp. However, combinations of SAHA with azoles showed synergistic effects against most planktonic cells and biofilms. This effect was most noticeable when SAHA was combined with ITR or VRC, although there were weak synergistic effects between SAHA and POC. In addition, SAHA was able to strengthen the antifungal effects of azoles. The half-maximal effective concentration (EC50) of SAHA against another human pathogen, *Cryptosporidium parvum,* has been reported as 0.203 μM [17]. Despite of a lack of inhibitory effects of SAHA against *Aspergillus*, the synergistic effects observed with the azole combination indicate that the MICs of SAHA may be acceptable for in vivo treatment. The effective doses of SAHA (400 mg/day oral dose for adults) would be able to reach the effective concentration that, with the addition of azoles, could make it appropriate for the treatment of aspergillosis. The mechanisms of resistance of *A. fumigatus* biofilms to antifungal drugs are strongly associated with genes encoding ABC transporters [6]. HSP90 is also involved in the resistance of *A. fumigatus* biofilms to drugs, and impairment of HSP90 function could reverse azole resistance in eradicating biofilms [18]. Furthermore, blocking the action of HSP90 increases the azole sensitivity of fungi [19]. To explain the probable synergistic effects of SAHA and azoles against *Aspergillus* or its biofilm, the expression of the azole susceptibility-related drug efflux pumps *MDR1*, *MDR2*, *MDR3*, and *MDR4,* as well as *HSP90* levels, was measured by RT-PCR. The results showed that the molecular mechanism of the observed SAHA synergistic effects with azoles in *A. fumigatus* biofilms might be associated with diminished expression of these efflux pumps. Although the diminished expression of efflux pump genes was comparatively mild in the combination groups compared with that in the azole-only groups (1.3- to 2.6-fold) and in the combinations of SAHA with VRC and POC, the expression of all four efflux pump genes showed a statistically significant reduction compared with that after azole treatment alone (Fig. 1). The precise effects of altered expression of these efflux pumps need to be further investigated by using different knockout strains. Inactivation of HDACs in *C. albicans* was observed to block the HSP90-dependent response involved in azole resistance and restore azole susceptibility [6]. In our study, by using TSA as a rescue agent, the FICIs of different combination groups showed no change, confirming that the main synergistic mechanism of SAHA with azoles was mediated by HDAC inhibition. HDACs deacetylate and control the activation of multiple cytosolic proteins, such as HSP90 [9]. TSA is able to inhibit both class 1 and class 2 HDACs, thus displaying antifungal activity against *A. fumigatus* and having a similar phenotype to genetic repression of *HSP90* [20]. In the present study, the expression levels of *HSP90* were significantly suppressed in the SAHA combination groups compared with those in the azole-only groups. This suppression of *HSP90* was consistent with the reported actions of TSA, which supports the hypothesis that the synergistic effects of SAHA with azoles are mediated through *HSP90* inhibition. Further investigation of the relationship between *HSP90* and SAHA and their pathways in *Aspergillus* virulence and azole susceptibility may help to understand the mechanisms of the observed synergistic interactions. HDAC inhibitors have the potential to act as anticancer agents, which may motivate clinical as well as preclinical trials. There is thus a strong incentive to further research HDAC inhibitors, including SAHA, to gain insight into and understanding of the role of HDACs in fungi, as well as to investigate more effective therapeutic strategies against infections caused by *Aspergillus.*

## Conclusions

In the current study, investigation of the HDAC inhibitor SAHA demonstrated its potential as a secondary treatment to improve the effects of azole treatment against both biofilm and planktonic states of *Aspergillus spp.* This activity appears to occur largely via the inhibition of *HSP90*. Future in vivo studies as well as studies focusing on underlying mechanisms will be beneficial to understand the clinical use of these combinations against *Aspergillus*-associated infections.

## Methods

### Fungal strains

A total of 20 strains of *Aspergillus* spp. comprising twelve strains of *A. fumigatus* (AF102-AF1010 were isolated from IA patients; AF1011 and AF1012 were isolated from chronic rhinosinusitis patients), five strains of *A. flavus* (AFLA101-AFLA103 were isolated from IA patients; AFLA104 and AFLA105 were isolated from chronic rhinosinusitis patients), and three strains of *A. terreus* (AT101-AT103 were isolated from chronic rhinosinusitis patients) were studied. All the clinical strains were acquired from patients admitted to our hospital. All patients provided written informed consent. This study was approved by the patients as well as the Research Ethics Committee of the First Affiliated Hospital Jinan University. Both microscopic morphology and sequencing of the ITS were performed for fungal identification. *A. flavus* strain ATCC 204304 was included as a quality control.

### Patient information

AF102-AF1010 and AFLA101-AFLA103 were isolated from patients with acute leukemia with IA during systemic chemotherapy. AF1011, AF1012, AFLA104, AFLA105 and AT101-AT103 were isolated from patients with chronic rhinosinusitis without invasive infection.

### Antifungal and chemical agents

All tested drugs, including SAHA (purity ≥99%), itraconazole (ITR, purity ≥99%), voriconazole (VRC, purity ≥99%), posaconazole (POC, purity ≥99%) and TSA (purity ≥97%), were bought in powder form from Selleck Chemicals (Houston, TX, USA) and prepared as described in the Clinical and Laboratory Standards Institute (CLSI) broth microdilution method M38-A2 [21]. All the drugs were dissolved in DMSO to formulate stock solutions with a concentration of 6400 μg/ml. The working concentration ranges for antifungal tests and antifungal combinations of SAHA, ITR, VRC, and POC were 0.03–16 μg/ml for planktonic cells and 2–256 μg/ml for biofilms.

### Preparation of inoculums

Fresh conidia collected from Sabouraud dextrose agar (SDA) cultures were suspended in sterile distilled water containing 0.03% Triton. A hemocytometer was used to adjust the concentrations to 1–5 × 10^6^ spores/ml, which were then diluted 100 times in RPMI-1640 buffered with 165 mM morpholinepropanesulfonic acid to pH 7. The final densities of conidia were approximately 1–5 × 10^4^ spores/ml [11].

### Preparation of biofilms

*Aspergillus* biofilms were prepared as previously described [22]. Fresh conidia on SDA were collected and suspended in 20 ml of RPMI-1640 according to the supplier’s protocol. The cells were suspended in RPMI-1640 to a final concentration of 1.0 × 10^5^ cells/ml. Subsequently, 200 μl of the suspension was added to wells of 96-well plates and incubated at 37 °C for 48 h. After the maturation of the biofilms, the medium was carefully removed without disturbing the biofilm. The 96-well plates were then washed carefully three times with sterile PBS to remove detached spores [22].

### In vitro interactions of SAHA and azoles against planktonic forms

The microdilution chequerboard technique and CLSI broth microdilution method M38-A2 were used to evaluate the combinations of SAHA and azoles [11, 21]. Specifically, serial dilutions of 50 μl of SAHA and 50 μl of azoles were added in the horizontal and vertical directions, respectively, of the 96-well plates. The final volume of each well was 200 μl containing 100 μl of prepared inoculum suspension plus 100 μl of drugs. After incubation at 35 °C for 48 h, the MIC values of SAHA and azoles were recorded as the lowest concentration leading to complete inhibition of growth, as assessed visually. The FICI was used to classify the interactions of drug combinations. For the concentration of MICs used for the FICI calculation that was above the maximum concentration tested, we used double this value. The FICIs were calculated by MIC as previously described [11]. The FICI was classified as follows: FICI of ≤0.5, synergy; FICI of >0.5 to ≤4, no interaction; and FICI of >4, antagonism. All tests were performed in triplicate by biological replication [23].

### In vitro interactions of SAHA and azoles against biofilms

Using the checkerboard method, SAHA and azoles were added to 96-well plates containing the prepared biofilms. The XTT-based colorimetric assay was used. Briefly, azoles or SAHA were added first to biofilm-containing wells, followed by incubation at 37 °C for 24 h and subsequent addition of μl XTT/menadione solution to each well and further incubation for 2 h at 37 °C. Finally, 80 μl of the colored supernatant from each well was transferred into a new 96-well plate. This new 96-well plate was read at 490 nm to lessen the color influence of spores or biofilms. The SMIC80 was read as an 80% decrease in optical density compared to that of the controls. The interactions of SAHA with azoles against the biofilms also were analyzed by FICI, which was based on the SMIC80 [24]. All experiments were performed in triplicate by biological replication.

### Triple combinations with TSA

To determine whether the synergistic effects of SAHA with azoles are due to the inhibition of HDACs, we added triple combinations of drugs with a three-dimensional checkerboard technique based on CLSI M38-A2 [25]. SAHA and azoles (ITR, VRC and POC) were laid out according to the double combinations, followed by the addition of a single concentration (2 μg/ml) of TSA to each plate, from which we were able to determine the MIC of each agent alone and the combined effects on the same plate. The concentrations of SAHA, ITR, VRC, and POC ranged from 0.03–16 μg/ml. Quality controls were also included. After incubation for 48 h, the MICs were determined visually as the lowest concentration exhibiting total inhibition of growth.

### Quantification of gene expression by RT–PCR

One-step RT–PCR was performed on an ABI 7500 machine according to the manufacturer’s instructions. Primers for *HSP90, MDR1, MDR2, MDR3, MDR4* and β-tubulin (synthesized by Shanghai Samgon Biotech Co., Ltd.) [26, 27] are listed in Table 3. Approximately 1 × 10^6^ cells/ml AF293 cells were incubated on a shaker (200 rpm) at 37 °C for 10 h in RPMI-1640 medium. Subinhibitory concentrations of azoles alone (0.25 μg/ml for ITR, 0.125 μg/ml for VRC, and 0.125 μg/ml for POC) or in combination with SAHA (4 μg/ml) were added to the media, in addition to a drug-free control. The chosen doses of drugs were based on the antifungal susceptibility test; however, they did not directly correlate with the FICIs. TRIzol and acid-washed glass beads were used to extract total RNA. cDNA was synthesized by using TransScript II First-Strand cDNA Synthesis SuperMix (Transgen Biotec, Beijing, China). qRT–PCR was performed using TransScript II Green One-Step qRT-PCR SuperMix (Transgen Biotec, Beijing, China) according to the supplier’s protocol. Amplification was achieved under the following conditions: 50 °C for 5 min, 94 °C for 30 s, and 40 cycles of 95 °C for 30 s followed by 60 °C for 30 s. Dissociation curves were examined to rule out any nontarget amplification. Gene expression was evaluated using the 2^-ΔΔCt^ method, with β-tubulin as the reference gene [27].

**Table 3:** Primers used in this study

Primer	Sequence
*HSP90*	5′-TCATGATCGCTCCATGTTG-3′
	5′-GAGGAGCTCAACAAGACCAA-3’
*MDR1*	5′-GAACGCACCACGAGTTGATT-3’
	5′-CTGCAGATTGACCAGCTCGTAATA-3’
*MDR2*	5′-CTTGTTATCCGCCAATGTCTGTAGT-3’
	5′-GGAGGTAGAAAACAGCCTGACT-3’
*MDR3*	5′-CATCCTCATTCCCTTGCATATCGT-3’
	5′-TCAAGCTATAACCGCCCACATG-3’
*MDR4*	5′-TTGCTGGTGTTTGGTGAGTGA-3’
	5′-GCCTCCTGTTTGATAATGCTCTCA-3’
*β-tubulin*	5′-GCACGTGAAATTGTTGAAAGG-3’
	5′-CAGGCTGGCCGCATTG-3’

### Statistical analysis

RT-PCR values were expressed as the means and standard errors of the mean (error bars), and graphs were generated using GraphPad Prism (version 6). Statistical analysis was performed by multiple t tests. The results were considered statistically significant at *P<*0.05.

## Data Availability

The data that support the findings of this study are available from the corresponding author on request.

## Abbreviations

FICI: Fractional inhibitory concentration index
HDACs: Histone deacetylases
IA: Invasive aspergillosis
ITR: Itraconazole
MIC: Minimal inhibitory concentration
POC: Posaconazole
SAHA: Suberoylanilide hydroxamic acid
SDA: Sabouraud dextrose agar
SMIC: Sessile minimal inhibitory concentration
VRC: Voriconazole
XTT: 2,3-Bis-[2-methoxy-4-nitro-5-sulfophenyl]-2H-tetrazolium-5-carboxanilide

## References

[1] Bongomin Felix, Gago Sara, Oladele Rita, Denning David (2017). Global and Multi-National Prevalence of Fungal Diseases—Estimate Precision. Journal of Fungi.

[2] Vaezi A, Fakhim H, Javidnia J, Khodavaisy S, Abtahian Z, Vojoodi M (2018). Pesticide behavior in paddy fields and development of azole-resistant *Aspergillus fumigatus*: should we be concerned?. J Mycol Med.

[3] Fakhim H, Vaezi A, Dannaoui E, Sharma C, Mousavi B, Chowdhary A (2018). In vitro combination of voriconazole with micafungin against azole-resistant clinical isolates of *Aspergillus fumigatus* from different geographical regions. Diagn Microbiol Infect Dis.

[4] Abastabar M, Rahimi N, Meis JF, Aslani N, Khodavaisy S, Nabili M (2016). Potent activities of novel imidazoles lanoconazole and luliconazole against a collection of azole-resistant and -susceptible *Aspergillus fumigatus* strains. Antimicrob Agents Chemother.

[5] Vaezi A, Fakhim H, Arastehfar A, Shokohi T, Hedayati MT, Khodavaisy S (2018). *In vitro* antifungal activity of amphotericin B and 11 comparators against *Aspergillus terreus* species complex. Mycoses.

[6] Beauvais A, Latge JP. *Aspergillus* biofilm *in vitro* and *in vivo*. Microbiol Spectr. 2015;3(4). 10.1128/microbiolspec MB-0017-2015. 10.1128/microbiolspec.MB-0017-2015 26350307

[7] Brewer JH, Thrasher JD, Hooper D (2013). Chronic illness associated with mold and mycotoxins: is naso-sinus fungal biofilm the culprit?. Toxins (Basel).

[8] Loussert C, Schmitt C, Prevost MC, Balloy V, Fadel E, Philippe B (2010). *In vivo* biofilm composition of *Aspergillus fumigatus*. Cell Microbiol.

[9] Lamoth F, Juvvadi PR, Steinbach WJ (2015). Histone deacetylase inhibition as an alternative strategy against invasive aspergillosis. Front Microbiol.

[10] Garnaud C, Champleboux M, Maubon D, Cornet M, Govin J (2016). Histone deacetylases and their inhibition in *Candida* species. Front Microbiol.

[11] Sun Y, Gao L, He C, Wu Q, Li M, Zeng T (2017). Givinostat exhibits *in vitro* synergy with posaconazole against *Aspergillus* spp. Med Mycol.

[12] Pfaller MA, Rhomberg PR, Messer SA, Castanheira M (2015). *In vitro* activity of a Hos2 deacetylase inhibitor, MGCD290, in combination with echinocandins against echinocandin-resistant *Candida* species. Diagn Microbiol Infect Dis.

[13] Li X, Cai Q, Mei H, Zhou X, Shen Y, Li D (2015). The Rpd3/Hda1 family of histone deacetylases regulates azole resistance in *Candida albicans*. J Antimicrob Chemother.

[14] Duvic M, Talpur R, Ni X, Zhang C, Hazarika P, Kelly C (2007). Phase 2 trial of oral vorinostat (suberoylanilide hydroxamic acid, SAHA) for refractory cutaneous T-cell lymphoma (CTCL). Blood.

[15] Xu J, Sun J, Wang P, Ma X, Li S (2018). Pendant HDAC inhibitor SAHA derivatised polymer as a novel prodrug micellar carrier for anticancer drugs. J Drug Target.

[16] Gao M, Chen G, Wang H, Xie B, Hu L, Kong Y (2016). Therapeutic potential and functional interaction of carfilzomib and vorinostat in T-cell leukemia/lymphoma. Oncotarget.

[17] Guo F, Zhang H, McNair NN, Mead JR, Zhu G (2018). The existing drug vorinostat as a new lead against *Cryptosporidiosis* by targeting the parasite histone deacetylases. J Infect Dis.

[18] Robbins N, Uppuluri P, Nett J, Rajendran R, Ramage G, Lopez-Ribot JL (2011). Hsp90 governs dispersion and drug resistance of fungal biofilms. PLoS Pathog.

[19] Gao L, Sun Y, He C, Li M, Zeng T (2018). *In vitro* interactions between 17-AAG and azoles against *Exophiala dermatitidis*. Mycoses.

[20] Lamoth F, Juvvadi PR, Soderblom EJ, Moseley MA, Asfaw YG, Steinbach WJ (2014). Identification of a key lysine residue in heat shock protein 90 required for azole and echinocandin resistance in *Aspergillus fumigatus*. Antimicrob Agents Chemother.

[21] Institute CaLS. Reference method for broth dilution antifungal susceptibility testing of filamentous fungi. Approved Standard M38-A2. 2008.

[22] Pierce CG, Uppuluri P, Tristan AR, Wormley FL, Mowat E, Ramage G (2008). A simple and reproducible 96-well plate-based method for the formation of fungal biofilms and its application to antifungal susceptibility testing. Nat Protoc.

[23] Odds FC (2003). Synergy, antagonism, and what the chequerboard puts between them. J Antimicrob Chemother.

[24] Gao L, Sun Y (2015). *In vitro* interactions of antifungal agents and tacrolimus against *Aspergillus* biofilms. Antimicrob Agents Chemother.

[25] Yao L, Wan Z, Li R, Yu J (2015). *In vitro* triple combination of antifungal drugs against clinical *Scopulariopsis* and *Microascus* species. Antimicrob Agents Chemother.

[26] Lamoth F, Juvvadi PR, Fortwendel JR, Steinbach WJ (2012). Heat shock protein 90 is required for conidiation and cell wall integrity in *Aspergillus fumigatus*. Eukaryot Cell.

[27] Li SX, Song YJ, Jiang L, Zhao YJ, Guo H, Li DM (2017). Synergistic effects of tetrandrine with posaconazole against *Aspergillus fumigatus*. Microb Drug Resist.

